# Comparative evaluation of three proliferation markers, Ki-67, TOP2A, and RacGAP1, in bronchopulmonary neuroendocrine neoplasms: Issues and prospects

**DOI:** 10.18632/oncotarget.9747

**Published:** 2016-05-31

**Authors:** Elisa Neubauer, Ralph M. Wirtz, Daniel Kaemmerer, Maria Athelogou, Lydia Schmidt, Jörg Sänger, Amelie Lupp

**Affiliations:** ^1^ Institute of Pharmacology and Toxicology, Jena University Hospital, Friedrich Schiller University, Jena, Germany; ^2^ STRATIFYER Molecular Pathology GmbH Köln, Köln, Germany; ^3^ Department of General and Visceral Surgery, Zentralklinik Bad Berka, Bad Berka, Germany; ^4^ DEFINIENS AG, München, Germany; ^5^ Laboratory of Cytology and Pathology, Bad Berka, Germany

**Keywords:** Ki-67, topoisomerase 2 alpha, RacGAP1, lung neuroendocrine neoplasms, IHC/RT-qPCR

## Abstract

The classification of bronchopulmonary neuroendocrine neoplasms (BP-NEN) into four tumor entities (typical carcinoids (TC), atypical carcinoids (AC), small cell lung cancers (SCLC), large cell neuroendocrine lung carcinomas (LCNEC)) is difficult to perform accurately, but important for prognostic statements and therapeutic management decisions. In this regard, we compared the expression of three proliferation markers, Ki-67, Topoisomerase II alpha (TOP2A), and RacGAP1, in a series of tumor samples from 104 BP-NEN patients (24 TC, 21 AC, 52 SCLC, 7 LCNEC) using different evaluation methods (immunohistochemistry (IHC): Average evaluation, Hotspot evaluation, digital image analysis; RT-qPCR).

The results indicated that all three markers had increased protein and mRNA expression with poorer differentiation and correlated well with each other, as well as with grading, staging, and poor survival. Compared with Ki-67 and TOP2A, RacGAP1 allowed for a clearer prognostic statement. The cut-off limits obtained for Ki-67-Average (IHC) were TC-AC 1.5, AC-SCLC 19, and AC-LCNEC 23.5. The Hotspot evaluation generated equal to higher, the digital image analysis generally lower between-entity cut-off limits.

All three markers enabled a clear-cut differentiation between the BP-NEN entities, and all methods evaluated were suitable for marker assessment. However, to define optimal cut-off limits, the Ki-67 evaluation methods should be standardized. RacGAP1 appeared to be a new marker with great potential.

## INTRODUCTION

According to the National Cancer Institute and the World Health Organization (WHO), lung cancer is the most common death-causing cancer. This relationship is particularly true in the well-industrialized northern hemisphere, where lung cancer comprises 20–25% of the malignancies of neuroendocrine origin. The 2015 WHO classification system (first proposed in 1998 by Travis et al. [1]) classifies these bronchopulmonary neuroendocrine neoplasms (BP-NEN) into the less aggressive, well-differentiated, and low-grade typical carcinoids (TC), the intermediate-grade atypical carcinoids (AC), and the poorly-differentiated and aggressive large cell neuroendocrine lung carcinomas (LCNEC) and small cell lung cancers (SCLC) [1–5]. Correct diagnostic classification of these four tumor entities is of major clinical importance because the prognostic, diagnostic, and therapeutic consequences differ greatly between them. The current grading system for NEN of the lung and thymus is based on WHO and International Association for the Study of Lung Cancer (IASLC) criteria: The grade is mainly determined by counting the number of mitotic figures in 10 high-power fields (HPFs) in combination with the presence or absence of necrosis [5, 6]. Counting mitoses is a very time-consuming process with a high inter-observer variability and cytomorphological overlap among the BP-NEN entities is not uncommon. An additional and reliable cut-off-marker is still needed to distinguish TC from AC and, more importantly, AC from SCLC/LCNEC, during development of a pathologic diagnosis [4, 7–9]. The Ki-67 labeling index was proposed for this purpose. The index was recently added to the 2010 WHO classification for tumor grading (Ki-67: G1: ≤ 2%; G2: 3–20%; G3: > 20%) of gastroenteropancreatic neuroendocrine neoplasms (GEP-NEN). It has emerged as a gold standard and a predictor of prognosis. Index results are also used for patient management decisions, particularly when advanced disease is present [10, 11]. The clinical acceptance of Ki-67, when used for BP-NEN, is still increasing, and clinical use of Ki-67 is being discussed extensively in the literature. More comprehensive studies are needed for a complete integration of Ki-67 into the grading system of this tumor group [12]. A uniform evaluation system (manual counting, eyeballing, digital image analysis), inter-observer variability of manual immunohistochemical evaluation, and the correct cut-off values to distinguish the BP-NEN entities are issues for further study [8, 9, 12–14]. The 2015 WHO classification for pulmonary neuroendocrine tumors recommends only very rough Ki-67 limits (TC ≤ 5%, AC ≤ 20%, SCLC 50–100%, and LCNEC 40–80%) and calls for additional studies to generate more data [5].

We propose that Topoisomerase II alpha (TOP2A) is another proliferation marker that has potential to be used to distinguish BP-NEN entities. It is used to determine the proliferative tumor cell fraction in other tumor entities (e.g., breast cancer). The advantage of TOP2A compared with Ki-67 is related to its additional function as a predictive marker for subsequent therapies with topoisomerase inhibitors or anthracyclines [15, 16]. TOP2A used as a histopathologic marker for NEN entities has not been tested so far.

The Rac GTPase activating protein 1 (RacGAP1) is a previously unknown but very interesting proliferation marker; it is upregulated in many malignant tumors and is associated with a poor patient outcome [17–21]. This protein binds to the activated forms of RhoGTPases and induces GTP hydrolysis. It, thus, negatively regulates many Rho-mediated signals [21, 22]. Little is known about RacGAP1, except that it is involved in the cytokinesis, migration, cell motility, and transformation of tumor cells, and subsequent increased metastasis [21, 23–26]. The role of RacGAP1 in NEN and its clinical functions remain to be elucidated, but first study results suggest that it is correlated with Ki-67 expression [19]. In addition to allowing us to differentiate between the BP-NEN entities, TOP2A and RacGAP1 may provide new insights into the biological and molecular behavior of NEN.

**Figure 1:**
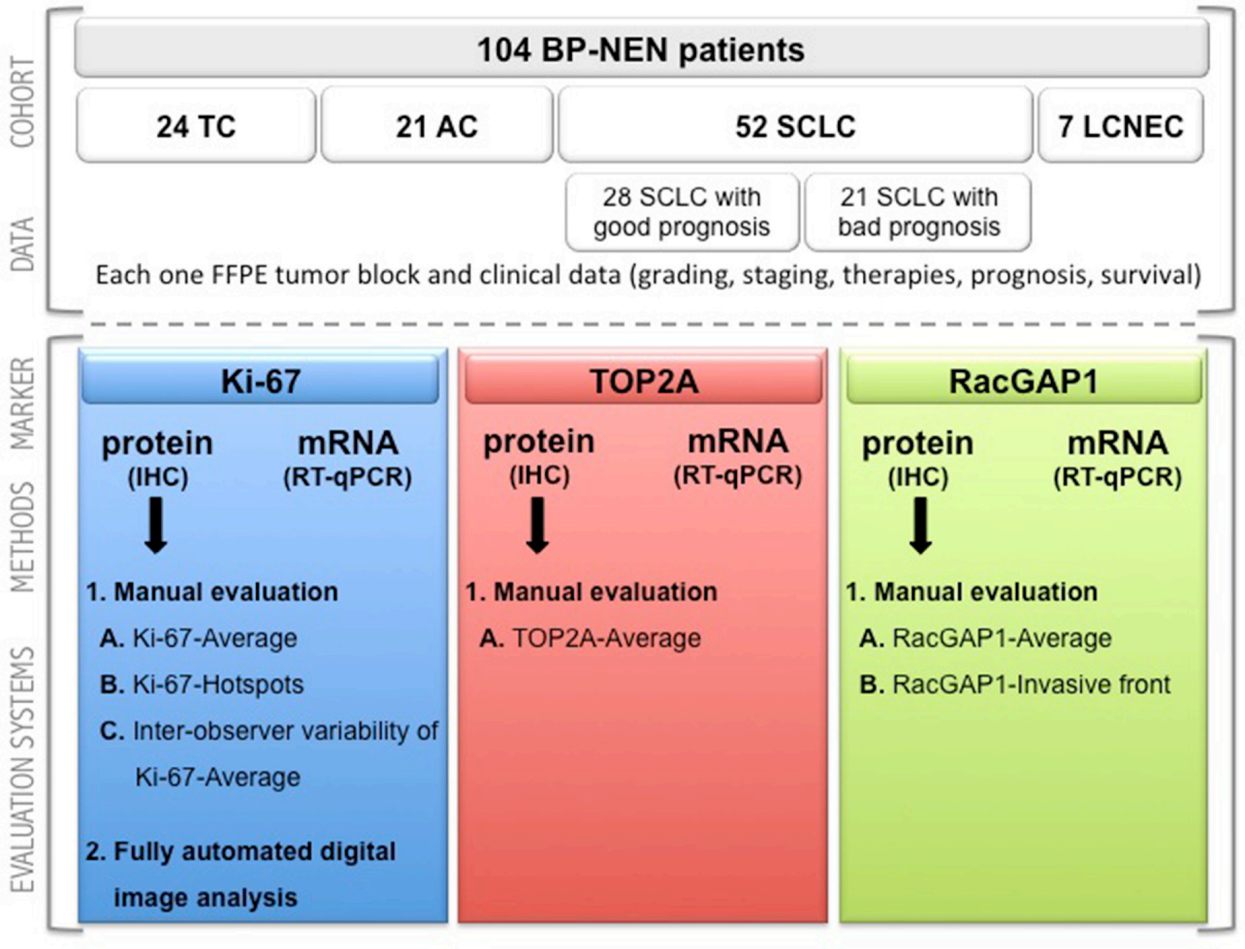
Consort diagram Overview of the patient population in terms of the investigated markers in the different evaluation methods. BP-NEN – bronchopulmonary neuroendocrine neoplasms; TC – typical carcinoids; AC – atypical carcinoids; SCLC – small cell lung cancer; LCNEC – large cell neuroendocrine lung carcinoma; FFPE – formalin-fixed, paraffin-embedded; TOP2A – Topoisomerase 2 alpha; RacGAP1 – Rac GTPase activating protein 1; IHC – immunohistochemistry.

The first aim of our study was to compare the current Ki-67 detemination methods when used for BP-NEN diagnosis. Two manual Ki-67 immunohistochemistry (IHC) evaluation methods were compared with a fully automated digital image analysis of the same slides and a real time RT-PCR of adjacent paraffin sections. The IHC inter-observer variability for the manual counting method was also evaluated. The second aim was to define Ki-67 cut-off values for the BP-NEN entities for each evaluation method. The Ki-67 protein and mRNA expression levels were then compared with those of TOP2A and RacGAP1. The suitability of the three proliferation markers for differentiation of the BP-NEN entities was then evaluated, and correlations with the patients' clinical data were calculated (overview over patient population see Figure 1).

## RESULTS

### Ki-67 expression in BP-NEN entities

### Immunohistochemistry – Ki-67-Average vs. Ki-67-Hotspots vs. digital image analysis

**Figure 2:**
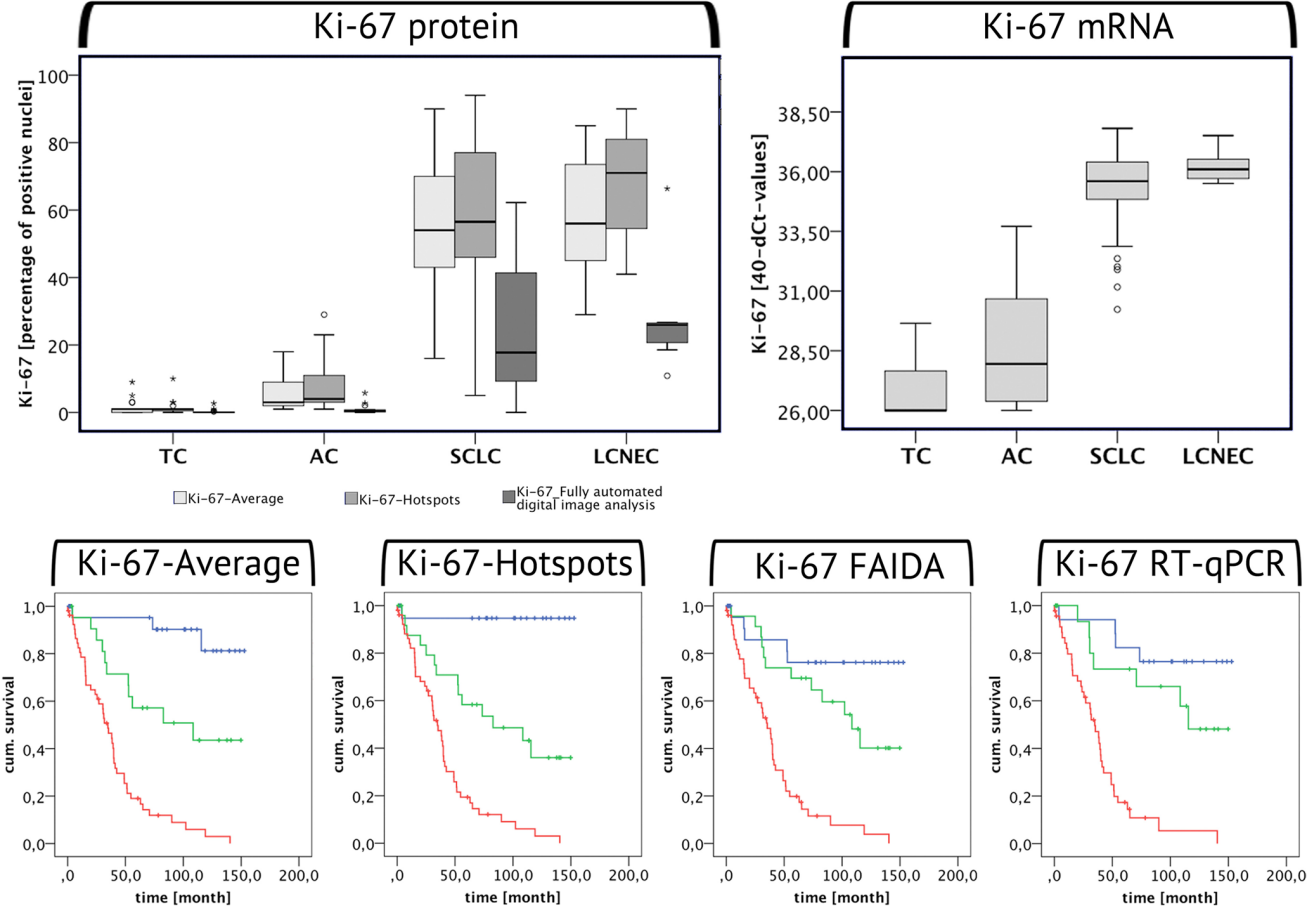
Ki-67 expression in BP-NEN The boxplots are depicting the Ki-67 protein (left) and mRNA (right) levels in the different BP-NEN entities as evaluated by immunohistochemistry and by RT-qPCR. The different Ki-67-IHC evlauation methods are represented by different shades of grey: The Ki-67-Average is depicted by the first box in light grey, the Ki-67-Hotspots are illustrated in slightly darker grey and the last dark grey box stands for the IHC Ki-67 levels as determined by the fully automated digital image analysis. Beneath the respective Kaplan-Meier-Analyses are shown. Blue indicates low, green moderate and red high Ki-67 expression levels in each evaluation method, according to the cut-off limits of the ROC analysis between TC – AC and AC – SCLC. High Ki-67 expression levels correlate with a poor survival of the patients.

As can be seen from the boxplots of Figure 2 and as confirmed by the Mann-Whitney-Test, Ki-67 protein levels increased highly significantly from TC to AC (Ki-67-Average: *U* = −4.337, *p* < 0.001, Ki-67-Hotspots: *U* = −5.041, *p* < 0.001; Ki-67-FADIA: *U* = −2.639, *p* = 0.008) and from AC to SCLC/LCNEC (Ki-67-Average: *U* = −6.592, *p* < 0.001/*U* = −3.913, *p* < 0.001, Ki-67-Hotspots: *U* = −6.277, *p* < 0.001/*U* = −3.921, *p* < 0.001; Ki-67-FADIA: *U* = −5.903, *p* < 0.001/*U* = −3.900, *p* < 0.001) in all three evaluation systems. The TC displayed very low data variability, whereas AC and especially SCLC and LCNEC showed a high spread of the proliferation marker protein levels.

It was found, that the evaluation of the Ki-67-Hotspots resulted in higher levels as compared to the use of the Ki-67-Average. A Wilcoxon-Test confirmed that these differences are significant in case of AC, SCLC and LCNEC, but not of TC. The Ki-67-FADIA generally led to lower Ki-67 expression levels compared with the two manual counting evaluation methods. According to the Wilcoxon-Test these differences are highly significant for TC, AC and SCLC (see Table 1). A similar tendency can be seen for LCNEC.

**Table 1:** Wilcoxon-test for Ki-67-Average vs. Ki-67-Hotspots vs. Ki-67-FADIA evaluation in BP-NEN entities

Tumor entity	BP-NEN total (*n* = 102)	TC (*n* = 24)	AC (*n* = 21)	SCLC (*n* = 50)	LCNEC (*n* = 7)
Ki-67-Hotspot < Ki-67-Average	8	1	0	7	0
Ki-67-Hotspots > Ki-67-Average	69	7	14	41	7
Ki-67-Hotspots = Ki-67-Average	25	16	7	2	0
*U*-value (Wilcoxon)	−5.662	−1.508	−3.310	−3.973	−2.371
*p*-value	< 0.001	0.132	0.001	< 0.001	0.018
Ki-67-FADIA < Ki-67-Hotspots	93	18	21	48	6
Ki-67- FADIA > Ki-67- Hotspots	8	5	0	2	1
Ki-67- FADIA = Ki-67- Hotspots	1	1	0	0	0
*U*-value (Wilcoxon)	−8.183	−3.224	−4.015	−6.115	−2.028
*p*-value	< 0.001	0.001	< 0.001	< 0.001	0.043
Ki-67-Average < Ki-67- FADIA	12	9	0	2	1
Ki-67- Average > Ki-67- FADIA	89	14	21	48	6
Ki-67- Average = Ki-67- FADIA	1	1	0	0	0
*U*-value (Wilcoxon)	−8.149	−2.829	−4.015	−6.125	−1.859
*p*-value	< 0.001	0.005	< 0.001	< 0.001	0.063

### Inter-observer comparability

Because the SCLC showed the highest spread of Ki-67 expression levels and the Ki-67-Average evaluation system is subjectively influenced at most, two different observers evaluated a series of 22 randomly selected SCLC samples for the Ki-67-Average in the IHC. In general, observer 1 (L.S.) obtained higher values (median = 72%, arithmetic mean = 65.7%) than observer 2 (E.N.) (median = 56, arithmetic mean = 56, 1%), as can be seen from the boxplots of Figure S1 in the Supplemental Data. Moreover, observer 1 obtained values with a higher spread and more outliers. However, these differences were not statistically significant (Wilcoxon-Test: *U* = −1.721, *p* = 0.085).

### RT-qPCR

Similar to the IHC, also the Ki-67 mRNA levels were highly significantly increased in AC compared to TC (*U* = −3.199, *p* = 0.001) and in SCLC/LCNEC compared to AC (U = −5.062, *p* < 0.001/*U* = −3.446, *p* < 0.001) (see Figure 2).

### Ki-67 cut-off values for grading

To detect the optimal Ki-67 cut-off points between TC and AC, AC and SCLC and AC and LCNEC, ROC analyses were performed. In our Ki-67-Average evaluation the optimal cut-off value to distinguish TC from AC is 1.5% (AUC = 0.870, *p* < 0.001, sensitivity = 0.810, 1-specificity = 0.167), AC from SCLC 19.0% (AUC = 0.998, *p* < 0.001, sensitivity = 0.960, 1-specificity = 0.000) and AC from LCENC 23.5% (AUC = 1.000, *p* < 0.001, sensitivity = 1.000, 1-specificity = 0.000). When evaluating the Ki-67-Hotspots the analysed cut-off value between TC and AC amounted to 1.5% (AUC = 0.930, *p* < 0.001, sensitivity = 0.952, 1-specificity = 0.167), between AC and SCLC to 22.0% (AUC = 0.974, *p* < 0.001, sensitivity = 0.900, 1-specificity = 0.095) and between AC and LCNEC to 35.00% (AUC = 1.000, *p* < 0.001, sensitivity = 1.000, 1-specificity = 0.000). In case of the Ki-67-FAIDA the cut-off values were much lower as compared to the manual Ki-67 evaluation methods: TC-AC: 0.14% (AUC = 0.730, *p* = 0.008, sensitivity = 0.762, 1-specificity = 0.208), AC-SCLC: 3.23% (AUC = 0.946, *p* < 0.001, sensitivity = 0.900, 1-specificity = 0.048), AC-LCNEC: 8.32% (AUC = 1.000, *p* < 0.001, sensitivity = 1.000, 1-specificity = 0.000). When analysing the RT-qPCR data, the following limits were obtained: TC-AC: 26.39 (AUC = 0.737, *p* = 0.021, sensitivity = 0.733, 1-specificity = 0.278), AC-SCLC: 32.19 (AUC = 0.977, *p* < 0.001, sensitivity = 0.907, 1-specificity = 0.067), AC-LCNEC: 34.61 (AUC = 1.000, *p* < 0.001, sensitivity = 1.000, 1-specificity = 0.000).

### TOP2A expression in BP-NEN entities

### Immunohistochemistry

**Figure 3:**
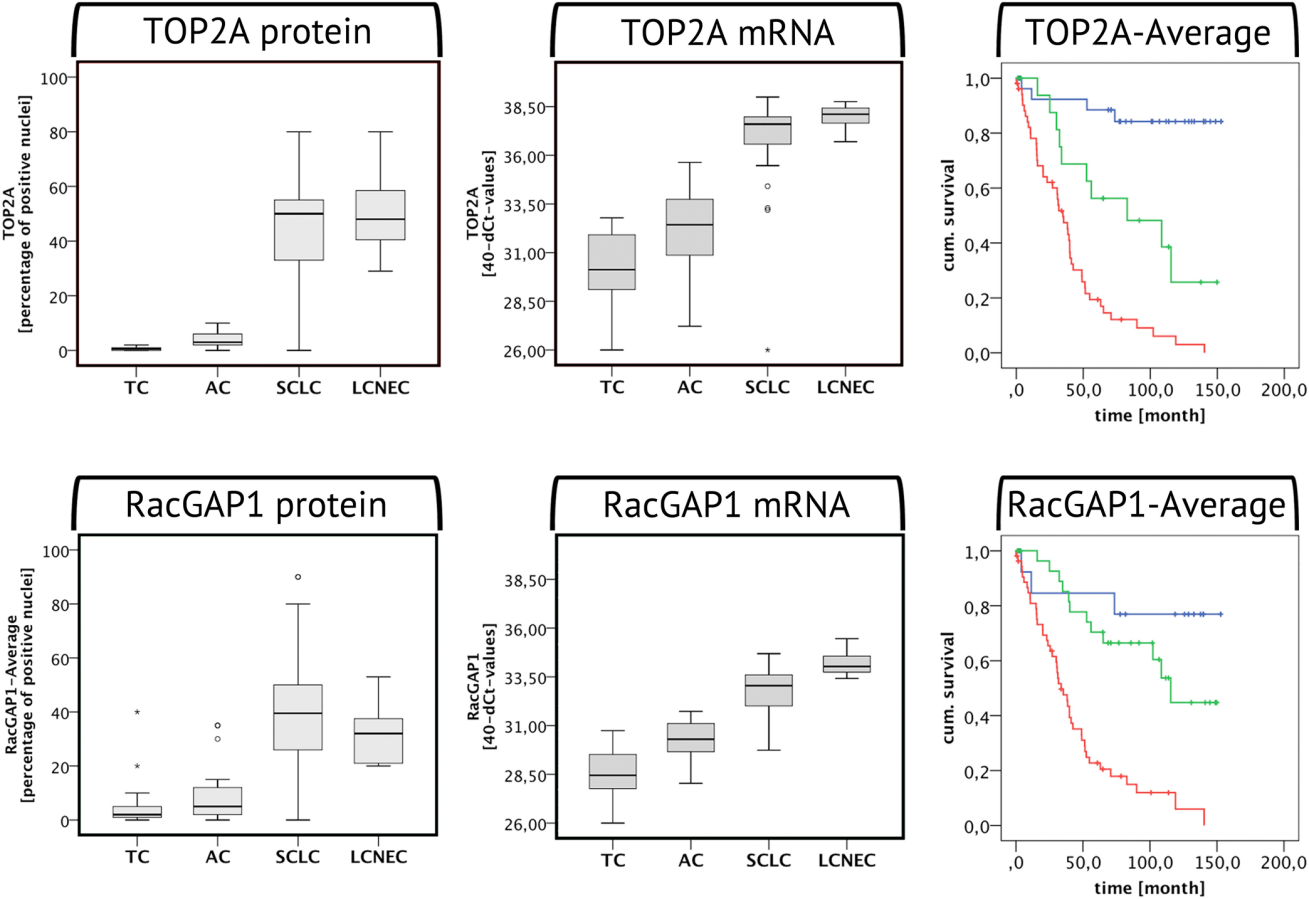
TOP2A and RacGAP1 expression in BP-NEN The boxplots are depicting the TOP2A and RacGAP1 protein (left) and mRNA (right) levels in the different BP-NEN entities as evaluated by immunohistochemistry and RT-qPCR. To the right the respective Kaplan-Meier-Analyses of each marker in the IHC investigation are shown. Blue indicates low, green moderate and red high TOP2A/RacGAP1 expression levels, according to the cut-off limits of the ROC analysis between TC – AC and AC – SCLC. With both markers, high expression levels imply poor patient survival.

In accordance with the Ki-67 expression levels, the TOP2A-Average protein expression significantly increased from TC to AC (Mann-Whitney-Test: *U* = −4.165, *p* < 0.001) and from AC to SCLC and LCNEC (Mann-Whitney-Test: *U* = −6.376, *p* < 0.001/*U* = −3.811, *p* < 0.001) (see Figure 3). TOP2A protein expression highly significantly correlated with the Ki-67-Average protein expression (ρ_sp_ = 0.896, *p* < 0.001, *n* = 99). According to the ROC analysis, the cut-off values between the respective tumor entities for the TOP2A expression as determined by IHC were as follows: TC-AC: 1.25% (AUC = 0.896, *p* < 0.001, sensitivity = 0.810, 1-specificity = 0.042), AC-SCLC: 15.0% (AUC = 0.981, *p* < 0.001, sensitivity = 0.980, 1-specificity = 0.000), AC-LCNEC: 19.5% (AUC = 1.000, *p* < 0.001, sensitivity = 1.000, 1-specificity = 0.000).

### RT-qPCR

The TOP2A mRNA expression showed a highly significant correlation with the Ki-67 mRNA levels (Spearman's rank correlation: ρ_sp_ = 0.907, *p* < 0.001, *n* = 83). There were higher TOP2A expression levels in AC compared to TC (Mann-Whitney-Test: *U* = −3.359, *p* = 0.001) and in SCLC/LCNEC compared to AC (*U* = −4.537, *p* < 0.001/*U* = −3.266, *p* < 0.001) (see Figure 3). In the RT-qPCR the following limits were determined according to the ROC analysis: TC-AC: 31.21 (AUC = 0.780, *p* = 0.006, sensitivity = 0.733, 1-specificity = 0.278), AC-SCLC: 34.96 (AUC = 0.955, *p* < 0.001, sensitivity = 0.907, 1-specificity = 0.067), AC-LCNEC: 36.17 (AUC = 1.000, *p* < 0.001, sensitivity = 1.000, 1-specificity = 0.000).

### RacGAP1 expression in BP-NEN entities

### Immunohistochemistry – RacGAP1-Average

The RacGAP1 protein levels also increased from TC to AC (Mann-Whitney-Test: *U* = −2.495, *p* = 0.013) and from AC to SCLC/LCNEC (Mann-Whitney-Test: *U* = −5.705, *p* < 0.001/*U* = −3.404, *p* < 0.001) (see Figure 3). RacGAP1 protein expression highly significantly correlated with the Ki-67 (ρ_sp_ = 0.733, *p* < 0.001, *n* = 98) and with the TOP2A protein expression (ρ_sp_ = 0.739, *p* < 0.001, *n* = 98). The cut-off limits for RacGAP1-Average according to the ROC curve were determined as follows: TC-AC: 1.5% (AUC = 0.705, *p* = 0.019, sensitivity = 0.857, 1-specificity = 0.583), AC-SCLC: 13.5% (AUC = 0.897, *p* < 0.001, sensitivity = 0.875, 1-specificity = 0.238), AC-LCNEC: 17.5% (AUC = 0.918, *p* = 0.001, sensitivity = 1.000, 1-specificity = 0.143).

### RT-qPCR

In the RT-qPCR, RacGAP1 expression was highly significantly increased in AC as compared to TC (Mann-Whitney-Test: *U* = −4.119, *p* < 0.001) and in SCLC/LCNEC as compared to AC (Mann-Whitney-Test: *U* = −4.116, *p* < 0.001 / *U* = −3.535, *p* < 0.001) (see Figure 3). In case of RacGAP1 mRNA levels, there was a highly significant increase in LCNEC in comparison to SCLC (Mann-Whitney-Test: *U* = −3.374, *p* < 0.001). RacGAP1 mRNA expression levels showed a highly significant correlation with the respective levels of Ki-67 and TOP2A in a Spearman's rank correlation (RacGAP1 vs. Ki-67: ρ_sp_ = 0.894, *p* < 0.001, *n* = 83; RacGAP1 vs. TOP2A: ρ_sp_ = 0.898, *p* < 0.001, *n* = 83). In the RT-qPCR the following limits were obtained according to the ROC analysis: TC-AC: 29.62 (AUC = 0.863, *p* < 0.001, sensitivity = 0.800, 1-specificity = 0.167), AC-SCLC: 31.52 (AUC = 0.933, *p* < 0.001, sensitivity = 0.837, 1-specificity = 0.067), AC-LCNEC: 32.58 (AUC = 1.000, *p* < 0.001, sensitivity = 1.000, 1-specificity = 0.000).

### Correlation of Ki-67, TOP2A and RacGAP1 with the mitotic rate

All markers (Ki-67-Hotspots, Ki-67-Average, TOP2A-Average, RacGAP1-Average, Ki-67 mRNA TOP2A mRNA, RacGAP1 mRNA) displayed a highly significant correlation with the mitotic rate as evaluated by two independent pathologists (ρ_sp_ = 0.667–0.889, *p* < 0.001–0.009). According to the Spearman's rank correlation only the Ki-67-FADIA showed no significant association with the mitotic count (ρ_sp_ = 0.399, *p* = 0.101).

### Correlation of Ki-67, TOP2A and RacGAP1 with clinical data and extent of necrosis

### Grading, staging and prognosis

Strong positive correlations with the grading and the T and N stage of the BP-NEN patients investigated could be observed for all three proliferation markers both in IHC and in RT-qPCR (see Table 2). When comparing SCLC patients with good and poor prognosis, solely RacGAP1 protein levels were significantly elevated in patients with poor prognosis.

**Table 2:** Spearman's rank correlations of Ki-67, TOP2A and RacGAP1 protein and mRNA levels with clinical data and extent of necrosis

	Grading	T stage	*N* stage	Prognosis	Necrosis
Ki-67-Average (IHC)	ρ_sp_ = 0.861 *p* < 0.001 *n* = 96	ρ_sp_ = 0.498 *p* < 0.001 *n* = 78	ρ_sp_ = 0.581 *p* < 0.001 *n* = 79	ρ_sp_ = 0.064 *p* = 0.669 *n* = 47	ρ_sp_ = 0.595 *p* < 0.001 *n* = 102
Ki-67 RT-qPCR	ρ_sp_ = 0.826 *p* < 0.001 *n* = 79	ρ_sp_ = 0.508 *p* < 0.001 *n* = 65	ρ_sp_ = 0.619 *p* < 0.001 *n* = 66	ρ_sp_ = 0.087 *p* = 0.590 *n* = 41	ρ_sp_ = 0.483 *p* < 0.001 *n* = 83
TOP2A-Average (IHC)	ρ_sp_ = 0.836 *p* < 0.001 *n* = 95	ρ_sp_ = 0.446 *p* < 0.001 *n* = 78	ρ_sp_ = 0.589 *p* < 0.001 *n* = 79	ρ_sp_ = 0.271 *p* = 0.068 *n* = 46	ρ_sp_ = 0.599 *p* < 0.001 *n* = 101
TOP2A RT-qPCR	ρ_sp_ = 0.809 *p* < 0.001 *n* = 79	ρ_sp_ = 0.524 *p* < 0.001 *n* = 65	ρ_sp_ = 0.605 *p* < 0.001 *n* = 66	ρ_sp_ = 0.175 *p* = 0.273 *n* = 41	ρ_sp_ = 0.525 *p* < 0.001 *n* = 83
RacGAP1-Average (IHC)	ρ_sp_ = 0.721 *p* < 0.001 *n* = 94	ρ_sp_ = 0.364 *p* = 0.001 *n* = 78	ρ_sp_ = 0.384 *p* = 0.001 *n* = 79	ρ_sp_ =−0.346 *p* = 0.018 *n* = 46	ρ_sp_ = 0.479 *p* < 0.001 *n* = 100
RacGAP1 RT-qPCR	ρ_sp_ = 0.800 *p* < 0.001 *n* = 79	ρ_sp_ = 0.498 *p* < 0.001 *n* = 65	ρ_sp_ = 0.519 *p* < 0.001 *n* = 66	ρ_sp_ = 0.135 *p* = 0.399 *n* = 41	ρ_sp_ = 0.574 *p* < 0.001 *n* = 83

### Extent of necrosis

All samples were evaluated for the extent of necrosis as a percentage of the tumor fraction. Here, the expression levels of all three markers in all methods used showed a highly significant correlation to the amount of necrosis, as can be seen from Table 2.

### Survival

Based on the cut-off limits for TC vs. AC and AC vs. SCLC obtained by means of the ROC analysis, all three proliferation markers were subdivided into low, moderate and high expression levels and Kaplan-Meier-Analyses were performed. All three proliferation markers displayed a strong prognostic value in all methods used (see Figures 2 and 3): high Ki-67, TOP2A and RacGAP1 protein and mRNA levels correlated with poor patient survival. These data were highly significant according to the Log-Rank and Breslow Test.

**Figure 4:**
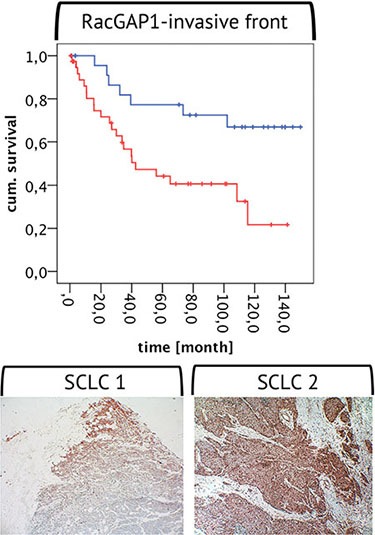
RacGAP1 expression at the invasive front Below two examples for a strong positivity of RacGAP1 protein expression at the invasive front are shown (magnification 10 × 10). The graph above depicts a Kaplan-Meier-Analysis with a high RacGAP1 protein expression (red line) and a weak/no RacGAP1 expression (blue line) at the invasive front. According to the Log-Rank Test the curves differ highly significantly: High RacGAP1 expression at the invasive front indicates poor patient prognosis.

### Proliferation marker expression in regard to chemotherapy and radiotherapy

Prior to tumor resection or biopsy, 17 patients (1 TC, 4 AC and 12 SCLC patients) had received a chemotherapy and 12 patients (1 TC, 2 AC and 9 SCLC) a radiotherapy. The tumor samples of these patients were compared with the samples from non-treated patients for their respective Ki-67, TOP2A and RacGAP1 mRNA and protein levels as determined by means of the different evaluation methods. According to the Mann-Whitney-Test for all three proliferation markers no marked difference was seen between the two subgroups, regardless of the method of determination. An exception was found in regard to the investigated SCLC patients: Here, the Ki-67-FADIA lead to significantly lower Ki-67 levels in patients with both previous radiotherapy and chemotherapy compared to the untreated SCLC patients (Mann-Whitney-Test: previous chemotherapy vs. untreated group: *U* = −2.680, *p* = 0.007; previous radiotherapy vs. untreated group: *U* = −1.957, *p* = 0.050) (see Figure S2 – Supplements). There was neither a difference to be seen between the two groups of patients for the Ki-67-Average nor for the Ki-67-Hotspot evaluation.

### Metastases vs. primary tumors

In the IHC investigations, samples of 87 patients with primary tumors and of 17 patients with metastases were included. Here, regardless of the method of determination, no differences in the protein expression levels of Ki-67, TOP2A or RacGAP1 were found between primary tumors and metastases. With regard to the mRNA levels, which were determined in 72 patients with primary tumors and in 11 patients with metastases, in contrast, the expression levels of Ki-67 were distinctly higher in metastases compared to primary tumors (Mann-Whitney-Test: *U* = −1.969, *p* = 0.049) and there was also a trend towards an increased expression of RacGAP1 (Mann-Whitney-Test: *U* = −1.847, *p* = 0.065) and TOP2A (Mann-Whitney-Test: *U* = −1.793, *p* = 0.073) in metastases (see Figure S3 – Supplements).

### Comparison between SCLC with good and with poor prognosis

In a cohort of 49 out of the 52 SCLC patients, comprising subjects with good prognosis (*n* = 28, survival ≥ 30 months) and with poor prognosis (*n* = 21, survival < 30 months) the three markers were investigated for their respective expression levels (see Figure S4 – Supplements). Here, RacGAP1 protein levels were significantly higher in patients with poor prognosis compared to those with good prognosis (Mann-Whitney-Test: *U* = −2.323, *p* = 0.020), but there was no difference on the mRNA levels (Mann-Whitney-Test: *U* = −0.855, *p* = 0.392). Ki-67 and TOP2A, in contrast, displayed no differences between the two subgroups of patients both on the protein and on the mRNA level (Mann-Whitney-Test: Ki-67: *U* = −0.434, *p* = 0.664/*U* = −0.548, *p* = 0.584; TOP2A: *U* = −1.819, *p* = 0.069/*U* = −1.109, *p* = 0.267).

### RacGAP1 as strong predictor of prognosis – expression at the invasive front

A high RacGAP1 expression at the invasive front of the selected tumor samples with resection margin significantly correlated with a high grading (ρ_sp_ = 0.494, *p* < 0.001, *n* = 59), with metastatic disease (ρ_sp_ = 0.337, *p* = 0.022, *n* = 46) and with early death by the tumor (ρ_sp_ = 0.279, *p* = 0.027, *n* = 63). According to the Kaplan-Meier-Analysis, patients with high RacGAP1 expression levels at the invasive front displayed lower survival as compared to those with low expression levels (see Figure 4).

## DISCUSSION

The distinction between the four BP-NEN entities is clinically very important because the subsequent classification-based diagnostic and therapeutic approaches differ due to their differential aggressiveness [3, 5, 6, 29, 30]. Mitosis counting is part of the classification procedure (based on the WHO and IASLC criteria); it is a very time-consuming process, and has a relatively high inter-observer variability [6, 8, 9]. The usefulness and limitations of Ki-67 as a marker for BP-NEN grading and differentiation are still being discussed. The 2015 WHO classification for pulmonary neuroendocrine tumors only suggests broad ranges for the Ki-67 labeling index in each entity. Therefore, one aim of our study was to reevaluate Ki-67 expression in the tumor samples of a large cohort of BP-NEN patients. The different methods routinely used in clinical practice for the determination of Ki-67 expression levels were compared in this context [5, 12].

The 2014 review by Pelosi et al. describes the many different immunohistochemical evaluation systems used for Ki-67 measurement [8, 9, 12–14, 31, 32]. In the present study, we compared two widely used manual counting methods, the Ki-67-Average (magnification 400×, per 10 representative HPFs) and the Ki-67-Hotspot evaluation (magnification 400×, per 2000 cells), with each other and with a fully automated digital image analysis of Ki-67 IHC. With all three methods, the Ki-67 expression significantly increased from TC to AC similar to previously published results [33]; the greatest increase occurred using Ki-67-Hotspot evaluation and the smallest increase occurred when the Ki-67-FADIA was used. There were also high and comparable expansions in the Ki-67 expression rates from AC to SCLC/LCNEC for each of the three methods. In the literature it is often described that Ki-67 mainly helps to distinguish low-grade from high-grade BP-NEN [12, 30], but our results also revealed that there was an increase in AC compared with TC. As expected, compared with the Ki-67-Average evaluation, the Ki-67 levels were higher in the Hotspot evaluation in cases of AC, SCLC, and LCNEC, but not TC. AC and, especially, SCLC have broader proliferation intensity ranges, and these more aggressive tumor entities often have one or more highly proliferative areas [8, 34, 35]. The three IHC evaluation methods were highly significantly correlated, but in general, lower proliferation rates were obtained for all tumor entities for Ki-67-FADIA. Here, between the tumor entities, a similar constellation was found as in both manual counting methods. The data are in accordance with many other studies comparing automated digital image analysis with the manual methods, but in some investigations also higher or equal limits were observed [31, 33, 36–38]. The discrepancies between published results may result from differences in the programs used for digital image analysis. Lower values may result from observer bias (i.e., a greater emphasis on positive compared with negative cells), whereas the FADIA allows for an objective evaluation. Furthermore, in our study either 2000 cells or 10 HPFs of a tumor sample were evaluated using the manual counting methods, whereas the FADIA included the whole tumor section. Besides, in the Ki-67-FADIA SCLC patients who underwent prior radiotherapy or chemotherapy had significantly lower Ki-67 index values compared with the untreated SCLC patients. The manual counting methods did not distinguish between these groups. To the best of our knowledge, we are the first group of investigators to perform a digital image analysis of Ki-67 expression in SCLC samples. We performed this sophisticated analysis even though nuclear shape was undefined and crushing artifacts were frequent. Our results appear to be valid, however, because they were consistent with the results using the other Ki-67 evaluation methods.

As often described, the eyeballing methods are subjectively influenced [8, 9, 12, 35]. Therefore, we also included an inter-observer comparison for the Ki-67 Average in 22 randomly selected SCLC patients using two independent observers. For this purpose we chose the SCLC patients because they had the widest range in proliferation rate and the Ki-67 Average evaluation because it displayed the largest relative error. Our study revealed that differences were present, but were not significant. Most authors report that compared with Ki-67 IHC evaluation, inter-observer variability is higher using mitotic counts [8, 9].

All manual counting methods revealed equal cut-off-limits between TC and AC and had good concordance with published results (approximately 2%). Compared with the Ki-67-Hotspot method, the cut-off values that discriminated between AC and SCLC/LCNEC were lower using Ki-67-Average. These limits are frequently discussed and debated in the literature [12, 33], but it is important to recognize that they are methodology-dependent. Our limits using Ki-67-Average were approximately 20% and our limits using Ki-67-Hotspot evaluation were approximately 25%. Nevertheless, our cut-off values were similar to previously established values for GEP-NEN grading [10, 39–43].

RT-qPCR is an emerging objective and reproducible method. Ki-67 mRNA has good clinical value when used for breast cancers, but comprehensive data are needed to determine the clinical value when used for NEN [16]. There were very significant correlations between protein and mRNA levels within adjacent paraffin sections for each of the three markers. We strongly recommend this method to distinguish between BP-NEN entities in routine pathology. However, more studies are needed to further strengthen our suggested limits and to define the correct cut-off values.

The third objective of our study was to investigate other markers that are clinically useful also for discrimination of NEN entities. We chose TOP2A because it is used for breast cancer diagnostics and because, as a target for anthracyclines and topoisomerase II inhibitors, it also has a direct predictive therapeutic value in many cancers [16, 44–47]. Our study revealed that TOP2A was significantly correlated with Ki-67 in our BP-NEN samples. This result occurred for protein and mRNA levels. TOP2A also increased with loss of differentiation, T stage, and grading. Therefore, we propose that TOP2A has clinical value for BP-NEN diagnosis.

RacGAP1 is an as of yet largely unknown but very interesting marker. RacGAP1 also increased from the well- to the poorly-differentiated BP-NEN entities and was strongly correlated with the results for the TOP2A and Ki-67 protein and mRNA levels. These results indicate that RacGAP1 can also be used as a proliferation marker [19]. The Kaplan-Meier-Analysis revealed that similar to the results for Ki-67 and TOP2A, high RacGAP1 levels correlated with poor patient outcome. RacGAP1 expression levels were, in general, lower than those of the other two markers, but a high expression rate was present at the invasive front of some tumor samples. Similarly, a study by Saigusa et al. revealed that the presence of high RacGAP1 levels at the invasive front of gastric cancer is associated with poor patient prognosis [21]. RacGAP1 also has a significantly higher protein expression rate in SCLC patients with a poor prognosis, compared with those with a good prognosis. This difference allows for a prognostic statement with more clarity, compared with the other two markers. The differences between the protein and mRNA levels are likely due to regulation by microRNAs [48, 49]. RacGAP1 is involved in cytokinesis, cell transformation, invasion, migration, and differentiation; it is also involved in tumorigenesis, and tumor progression, metastasis, and recurrence [19, 50–54]. It acts via mechanisms that are very complex and largely unknown. RacGAP1 is overexpressed in many very aggressive cancer phenotypes (e.g., meningiomas, hepatocellular carcinomas, epithelial ovarian cancer, invasive cervical cancer, high-grade breast cancer, colorectal cancer, non-small cell lung cancer, and gastric cancer [16, 18–21, 54–59]), which suggests that it is a new marker with great potential.

All in all, the expression levels of the three markers increase with indicators of tumor aggressiveness, including the T stage, the N stage and the extent of necrosis. A higher proliferation rate increases the risk for a higher extent of tumor growth, lymphatic invasion and the formation of necrosis. Thus, proliferation remains a key role in tumorigenesis and tumor progress.

In conclusion, we propose that Ki-67 should be used as an additional tool for BP-NEN grading and discrimination, with the Ki-67-FADIA as an objective IHC evaluation method. The Ki-67 IHC manual counting methods are also equally useful. They can be performed quickly and without the need for additional equipment. Here, standardization is needed to establish valid cut-off limits. The cut-off values we obtained for the manual Ki-67 counting method are similar to the limits used for GEP-NEN grading [43].

mRNA quantification using real time RT-PCR appears to be a very valid and objective alternative Ki-67 evaluation method. This approach should be investigated using a larger cohort of BP-NEN patients. Both on protein and on mRNA levels, TOP2A and RacGAP1 can also be used for histopathological differentiation of BP-NEN. RacGAP1 appears to have a strong prognostic value and is an interesting marker that should be examined in detail in future studies.

## MATERIALS AND METHODS

### Patients

Formalin-fixed, paraffin-embedded samples (FFPE) of primary tumors or metastases from a total of 104 patients, 24 of which diagnosed with a TC, 21 with an AC, 52 with a SCLC and 7 with a LCNEC, were provided by the Laboratory of Cytology and Pathology, Zentralklinik Bad Berka (Germany). The SCLC patients were further subdivided into patients with a good prognosis (*n* = 28, survival ≥ 30 months) and those with a poor prognosis (*n* = 21, survival < 30 months) (see Figure 1). All tumors were histologically verified by two independent pathologists according to the WHO and UICC criteria. The samples consisted mainly of tumor resections, but also some biopsy samples were included. They comprised mainly primary tumors, but some metastases were evaluated, too. 17 patients had received a previous chemotherapy and 12 patients a previous radiotherapy. A positive vote from the Ethics Committee of the state of Thuringia was gained for this retrospective analysis. Clinical data were collected from the patients records (Zentralklinik Bad Berka, Germany) and from the tumor registry data base (Tumorzentrum Helios-Klinik Erfurt, Germany).

### Immunohistochemistry

From each FFPE (104 samples from 104 BP-NEN patients) 4 μm thick sections were cut and floated onto positively charged slides. The immunohistochemistry was performed by an indirect peroxidase labeling method as described previously [27]. After dewaxing and microwaving of the sections in 10 mM citrate buffer (pH = 6.0) for 16 minutes at 600 W, the slices were incubated overnight at 4°C with the primary antibodies (Ki-67 (mouse monoclonal, 1:50, Dako Germany GmbH, Hamburg, GER), TOP2A (rabbit monoclonal, 1:100, Abcam, Cambridge, UK), RacGAP1 (rabbit polyclonal, 1:350, Atlas Antibodies, Stockholm, SWE)).

**Figure 5:**
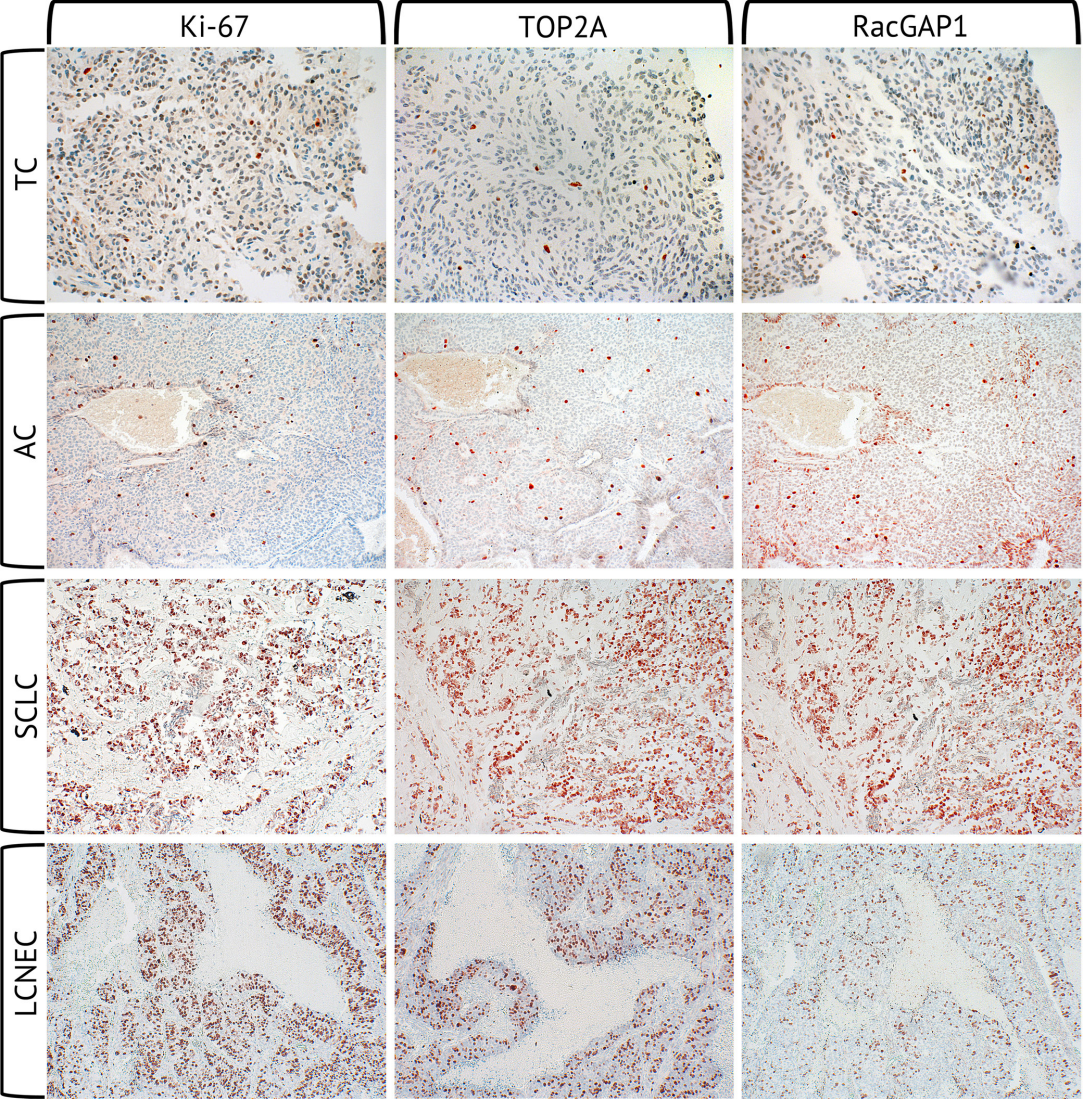
Immunohistochemical stainings of Ki-67, TOP2A and RacGAP1 Immunohistochemical staining of Ki-67, TOP2A and RacGAP1 at the same location of each one TC, AC, SCLC and LCNEC sample (magnification 40 × 10).

The detection was conducted with a biotinylated secondary antibody, followed by the addition of peroxidase-coupled avidin (VECTASTAIN^®^ Elite ABC kit, Vector Laboratories, Burlingame, CA, USA). The chromogen used was 3-amino-9-ethylcarbazole (AEC) (AEC substrate Pack, BioGenex, Fremont, CA, USA). Finally, cell nuclei were counterstained with Mayer's hematoxylin. Representative immunohistochemical stainings for each marker and tumor entity are shown in Figure 5.

### Manual evaluation methods of the immunohistochemistry

The IHC evaluation was performed by light microscopy. With all three markers, a nuclear staining of tumor cells was defined as a positive expression. Some markers were evaluated by different methods, as follows:

### Ki-67-Average, TOP2A-Average, RacGAP1-Average

At a magnification of 10 × 40 the total number of cell nuclei and the number of Ki-67, TOP2A or RacGAP1 positive nuclei, respectively, in 10 HPFs were counted manually and then the percentage of positive nuclei was calculated. Here, 10 representative HPFs were selected to determine the average proliferation rate of one tumor sample. In samples smaller than 10 HPFs, all cells were counted. In case of Ki-67, in a series of 22 tumor samples two different observers (E.N. and L.S.) independently evaluated the Ki-67-Average to assess the inter-observer variability.

### Ki-67-Hotspots

In each sample, the tumor region with the highest density of Ki-67 positive nuclei was selected and, here, the number of Ki-67 positive nuclei in a total of 2000 cells was counted manually at a magnification of 10 × 40. Finally, the percentage of Ki-67 positive nuclei was calculated.

### RacGAP1-invasive front

In samples with resection margin (*n* = 66) the front edge between the tumor and the surrounding tissues was defined as the invasive front, where tumor cells migrate into the neighboring tissue. Here, in comparison to the whole tumor and to the surrounding normal tissue, the RacGAP1 expression intensity was evaluated in four gradations: 0 – no RacGAP1 expression, 1 – weak RacGAP1 expression, 2 – moderate RacGAP1 expression, 3 – strong RacGAP1 expression at the invasive front. For further statistical analysis these data were classified into no/weak (0 and 1) and high (2 and 3) RacGAP1 expression at the invasive front.

### Fully automated digital image analysis (FADIA) of Ki-67 immunohistochemistry

All Ki-67 stainings were scanned at a magnification of 20× by means of a Hamamatsu digital slide scanner (NanoZoomer 2.0-HT, Hamamatsu Photonics, Japan). In cooperation with Definiens AG, Munich, Germany, the whole tumor areas of the tumor samples were fully automated analyzed by digital image analysis (Definiens Tissue Studio^®^) for the total number of tumor cell nuclei and for the Ki-67 positive tumor cell nuclei as previously described [28]. From these data, the percentage of Ki-67 positive nuclei was calculated.

### Real time RT-PCR

The adjacent 4-μm-paraffin sections of the immunohistochemical investigations of randomly selected 17 TC, 19 AC, 41 SCLC (13 with poor prognosis and 24 with good prognosis) and 7 LCNEC were analyzed for mRNA expression by a fully automated procedure in cooperation with STRATIFYER Molecular Pathology GmbH, Cologne. The mRNA was measured to draw conclusions on the proliferation marker expression at the transcriptional level. All FFPE were dewaxed and incubated with lysis buffer for 30 minutes. Here, the enzyme Proteinase K, which is contained in the buffer, leads to an enzymatic digestion of cellular components and proteins. After addition of MagiX^®^ RNA buffer, the extracted nucleic acids were bound to paramagnetic beads and the supernatant was pipetted off. Three washing steps followed. Ultimately, an elution buffer was added to release the nucleic acids from the beads. Finally, in the received eluate of nucleic acids the DNA was digested by the enzyme DNase I. All buffers used for the extraction are contained in the Extraction kit-XL (96)^®^ RNA 2.0 (STRATIFYER Molecular Pathology, Cologne, Germany).

Also fully automated, a multiplex TaqMan^™^-real-time PCR was performed after the extraction step. Here, the SuperScript^®^ III Platinum^®^ One-Step qRT-PCR kit and the Platinum Taq DNA polymerase (Invitrogen, Carlsbad, CA, USA) were used. All primers and probes were designed by STRATIFYER Molecular Pathology (Cologne, Germany) and manufactured by Eurogentec (Seraing, Belgium). CALM2 (calmodulin 2) served as a housekeeping gene and, in addition, a NTC (No Template Control) and a human reference RNA (QRef, Agilent Technologies, Mark Roeder, Germany) were determined. All samples were measured on a Mx3005P with the software “MxPro version 4.10d”. After 40 cycles (50 min 30°C, 2 min 95°C, (15 sec 95°C, 45 sec 60°C) × 40)), a logarithmic analysis at a threshold of 200 was conducted.

### Evaluation of the real time RT-PCR

The values measured were normalized by taking the difference between the Ct-value of the respective marker and the Ct-value of the housekeeping gene (CALM2), subtracted from the maximum number of PCR cycles (dCt(norm) = 40 − ΔCt (Ct(marker) - Ct(CALM2))). Thus, dCt-values ≥ 26.00 were obtained, which were used for further calculations. DCt-values ≥ 26.01 were classified as positive.

### Statistics

For statistical analysis the program IBM^®^ SPSS^®^ Statistics version 22.0.0 was used. According to the Kolmogorov-Smirnov test, the parameters were not normally distributed. Therefore, as statistical tests the Spearman's rank correlation, the Mann-Whitney-Test, the Wilcoxon-Test and the Kaplan-Meier-Analysis were performed. To evaluate the optimal cut-off values ROC analyses were performed. *P*-values < 0.05 were considered statistically significant and *p*-values < 0.01 highly significant.

## SUPPLEMENTARY FIGURES



## References

[1] Travis WD, Rush W, Flieder DB, Falk R, Fleming MV, Gal AA, Koss MN (1998). Survival analysis of 200 pulmonary neuroendocrine tumors with clarification of criteria for atypical carcinoid and its separation from typical carcinoid. Am J Surg Pathol.

[2] Gustafsson BI, Kidd M, Chan A, Malfertheiner MV, Modlin IM (2008). Bronchopulmonary neuroendocrine tumors. Cancer.

[3] Hörsch D, Sayeg Y, Bonnet R, Kaemmerer D, Presselt N, Baum RP (2012). [Expert dialogue: neuroendocrine tumours of the lungs and gastroenteropancreatic system]. Pneumologie.

[4] Siddiqui MT (2010). Pulmonary neuroendocrine neoplasms: a review of clinicopathologic and cytologic features. Diagn Cytopathol.

[5] Schnabel PA, Junker K (2015). [Pulmonary neuroendocrine tumors in the new WHO 2015 classification : Start of breaking new grounds?]. Pathologe.

[6] Phan AT, Oberg K, Choi J, Harrison LH, Hassan MM, Strosberg JR, Krenning EP, Kocha W, Woltering EA, Maples WJ, North American Neuroendocrine Tumor S (2010). NANETS consensus guideline for the diagnosis and management of neuroendocrine tumors: well-differentiated neuroendocrine tumors of the thorax (includes lung and thymus). Pancreas.

[7] Zheng G, Ettinger DS, Maleki Z (2013). Utility of the quantitative Ki-67 proliferation index and CD56 together in the cytologic diagnosis of small cell lung carcinoma and other lung neuroendocrine tumors. Acta Cytol.

[8] Swarts DR, van Suylen RJ, den Bakker MA, van Oosterhout MF, Thunnissen FB, Volante M, Dingemans AM, Scheltinga MR, Bootsma GP, Pouwels HM, van den Borne BE, Ramaekers FC, Speel EJ (2014). Interobserver variability for the WHO classification of pulmonary carcinoids. Am J Surg Pathol.

[9] Warth A, Fink L, Fisseler-Eckhoff A, Jonigk D, Keller M, Ott G, Rieker RJ, Sinn P, Soder S, Soltermann A, Willenbrock K, Weichert W, Pulmonary Pathology Working Group of the German Society of P (2013). Interobserver agreement of proliferation index (Ki-67) outperforms mitotic count in pulmonary carcinoids. Virchows Arch.

[10] Miller HC, Drymousis P, Flora R, Goldin R, Spalding D, Frilling A (2014). Role of Ki-67 Proliferation Index in the Assessment of Patients with Neuroendocrine Neoplasias Regarding the Stage of Disease. World J Surg.

[11] Pavel M, Kidd M, Modlin I (2013). Systemic therapeutic options for carcinoid. Semin Oncol.

[12] Pelosi G, Rindi G, Travis WD, Papotti M (2014). Ki-67 antigen in lung neuroendocrine tumors: unraveling a role in clinical practice. J Thorac Oncol.

[13] Pelosi G, Papotti M, Rindi G, Scarpa A (2014). Unraveling Tumor Grading and Genomic Landscape in Lung Neuroendocrine Tumors. Endocr Pathol.

[14] Rindi G, Klersy C, Inzani F, Fellegara G, Ampollini L, Ardizzoni A, Campanini N, Carbognani P, De Pas TM, Galetta D, Granone PL, Righi L, Rusca M (2014). Grading the neuroendocrine tumors of the lung: an evidence-based proposal. Endocr Relat Cancer.

[15] Jarvinen TA, Tanner M, Rantanen V, Barlund M, Borg A, Grenman S, Isola J (2000). Amplification and deletion of topoisomerase IIalpha associate with ErbB-2 amplification and affect sensitivity to topoisomerase II inhibitor doxorubicin in breast cancer. Am J Pathol.

[16] Milde-Langosch K, Karn T, Muller V, Witzel I, Rody A, Schmidt M, Wirtz RM (2013). Validity of the proliferation markers Ki67, TOP2A, and RacGAP1 in molecular subgroups of breast cancer. Breast Cancer Res Treat.

[17] Hazar-Rethinam M, de Long LM, Gannon OM, Boros S, Vargas AC, Dzienis M, Mukhopadhyay P, Saenz-Ponce N, Dantzic DD, Simpson F, Saunders NA (2015). RacGAP1 Is a Novel Downstream Effector of E2F7-Dependent Resistance to Doxorubicin and Is Prognostic for Overall Survival in Squamous Cell Carcinoma. Mol Cancer Ther.

[18] Imaoka H, Toiyama Y, Saigusa S, Kawamura M, Kawamoto A, Okugawa Y, Hiro J, Tanaka K, Inoue Y, Mohri Y, Kusunoki M (2015). RacGAP1 expression, increasing tumor malignant potential, as a predictive biomarker for lymph node metastasis and poor prognosis in colorectal cancer. Carcinogenesis.

[19] Ke HL, Ke RH, Li ST, Li B, Lu HT, Wang XQ (2013). Expression of RACGAP1 in high grade meningiomas: a potential role in cancer progression. J Neurooncol.

[20] Liang Y, Liu M, Wang P, Ding X, Cao Y (2013). Analysis of 20 genes at chromosome band 12q13: RACGAP1 and MCRS1 overexpression in nonsmall-cell lung cancer. Genes Chromosomes Cancer.

[21] Saigusa S, Tanaka K, Mohri Y, Ohi M, Shimura T, Kitajima T, Kondo S, Okugawa Y, Toiyama Y, Inoue Y, Kusunoki M (2015). Clinical significance of RacGAP1 expression at the invasive front of gastric cancer. Gastric cancer.

[22] Li J, Wang J, Jiao H, Liao J, Xu X (2010). Cytokinesis and cancer: Polo loves ROCK'n' Rho(A). J Genet Genomics.

[23] Burkard ME, Maciejowski J, Rodriguez-Bravo V, Repka M, Lowery DM, Clauser KR, Zhang C, Shokat KM, Carr SA, Yaffe MB, Jallepalli PV (2009). Plk1 self-organization and priming phosphorylation of HsCYK-4 at the spindle midzone regulate the onset of division in human cells. PLoS Biol.

[24] Petronczki M, Glotzer M, Kraut N, Peters JM (2007). Polo-like kinase 1 triggers the initiation of cytokinesis in human cells by promoting recruitment of the RhoGEF Ect2 to the central spindle. Dev Cell.

[25] Takaki T, Trenz K, Costanzo V, Petronczki M (2008). Polo-like kinase 1 reaches beyond mitosis—cytokinesis, DNA damage response, and development. Curr Opin Cell Biol.

[26] Wolfe BA, Takaki T, Petronczki M, Glotzer M (2009). Polo-like kinase 1 directs assembly of the HsCyk-4 RhoGAP/Ect2 RhoGEF complex to initiate cleavage furrow formation. PLoS Biol.

[27] Lupp A, Danz M, Muller D (2001). Morphology and cytochrome P450 isoforms expression in precision-cut rat liver slices. Toxicology.

[28] Nowak M, Svensson MA, Carlsson J, Vogel W, Kebschull M, Wernert N, Kristiansen G, Andren O, Braun M, Perner S (2014). Prognostic significance of phospho-histone H3 in prostate carcinoma. World J Urol.

[29] Travis WD (2010). Advances in neuroendocrine lung tumors. Ann Oncol.

[30] Sayeg Y, Sayeg M, Baum RP, Kulkarni HR, Presselt N, Mader I, Kunze A, Sanger J, Horsch D, Bonnet R (2014). [Pulmonary neuroendocrine neoplasms]. Pneumologie.

[31] Liu SZ, Staats PN, Goicochea L, Alexiev BA, Shah N, Dixon R, Burke AP (2014). Automated quantification of Ki-67 proliferative index of excised neuroendocrine tumors of the lung. Diagn Pathol.

[32] van Velthuysen ML, Groen EJ, van der Noort V, van de Pol A, Tesselaar ME, Korse CM (2014). Grading of neuroendocrine neoplasms: mitoses and Ki-67 are both essential. Neuroendocrinology.

[33] Joseph MG, Shibani A, Panjwani N, Arab A, Shepherd J, Stitt LW, Inculet R (2015). Usefulness of Ki-67, Mitoses, and Tumor Size for Predicting Metastasis in Carcinoid Tumors of the Lung: A Study of 48 Cases at a Tertiary Care Centre in Canada. Lung Cancer Int.

[34] Yamamoto S, Chishima T, Mastubara Y, Adachi S, Harada F, Toda Y, Arioka H, Hasegawa N, Kakuta Y, Sakamaki K (2015). Variability in measuring the Ki-67 labeling index in patients with breast cancer. Clin Breast Cancer.

[35] Singh S, Hallet J, Rowsell C, Law CH (2014). Variability of Ki67 labeling index in multiple neuroendocrine tumors specimens over the course of the disease. Eur J Surg Oncol.

[36] van Velthuysen ML, Groen EJ, Sanders J, Prins FA, van der Noort V, Korse CM (2014). Reliability of proliferation assessment by Ki-67 expression in neuroendocrine neoplasms: eyeballing or image analysis?. Neuroendocrinology.

[37] Fasanella S, Leonardi E, Cantaloni C, Eccher C, Bazzanella I, Aldovini D, Bragantini E, Morelli L, Cuorvo LV, Ferro A, Gasperetti F, Berlanda G, Dalla Palma P (2011). Proliferative activity in human breast cancer: Ki-67 automated evaluation and the influence of different Ki-67 equivalent antibodies. Diagn Pathol.

[38] Tang LH, Gonen M, Hedvat C, Modlin IM, Klimstra DS (2012). Objective quantification of the Ki67 proliferative index in neuroendocrine tumors of the gastroenteropancreatic system: a comparison of digital image analysis with manual methods. Am J Surg Pathol.

[39] Hentic O, Couvelard A, Rebours V, Zappa M, Dokmak S, Hammel P, Maire F, O'Toole D, Levy P, Sauvanet A, Ruszniewski P (2011). Ki-67 index, tumor differentiation, and extent of liver involvement are independent prognostic factors in patients with liver metastases of digestive endocrine carcinomas. Endocr Relat Cancer.

[40] Khan MS, Luong TV, Watkins J, Toumpanakis C, Caplin ME, Meyer T (2013). A comparison of Ki-67 and mitotic count as prognostic markers for metastatic pancreatic and midgut neuroendocrine neoplasms. Br J Cancer.

[41] Scarpa A, Mantovani W, Capelli P, Beghelli S, Boninsegna L, Bettini R, Panzuto F, Pederzoli P, delle Fave G, Falconi M (2010). Pancreatic endocrine tumors: improved TNM staging and histopathological grading permit a clinically efficient prognostic stratification of patients. Mod Pathol.

[42] Martin-Perez E, Capdevila J, Castellano D, Jimenez-Fonseca P, Salazar R, Beguiristain-Gomez A, Alonso-Orduna V, Martinez Del Prado P, Villabona-Artero C, Diaz-Perez JA, Monleon A, Marazuela M, Pachon V (2013). Prognostic factors and long-term outcome of pancreatic neuroendocrine neoplasms: Ki-67 index shows a greater impact on survival than disease stage. The large experience of the Spanish National Tumor Registry (RGETNE). Neuroendocrinology.

[43] Richards-Taylor S, Ewings SM, Jaynes E, Tilley C, Ellis SG, Armstrong T, Pearce N, Cave J (2015). The assessment of Ki-67 as a prognostic marker in neuroendocrine tumours: a systematic review and meta-analysis. J Clin Pathol.

[44] de Resende MF, Vieira S, Chinen LT, Chiappelli F, da Fonseca FP, Guimaraes GC, Soares FA, Neves I, Pagotty S, Pellionisz PA, Barkhordarian A, Brant X, Rocha RM (2013). Prognostication of prostate cancer based on TOP2A protein and gene assessment: TOP2A in prostate cancer. J Trans Med.

[45] Fountzilas G, Christodoulou C, Bobos M, Kotoula V, Eleftheraki AG, Xanthakis I, Batistatou A, Pentheroudakis G, Xiros N, Papaspirou I, Koumarianou A, Papakostas P, Bafaloukos D (2012). Topoisomerase II alpha gene amplification is a favorable prognostic factor in patients with HER2-positive metastatic breast cancer treated with trastuzumab. J Transl Med.

[46] Lukka PB, Chen YY, Finlay GJ, Joseph WR, Richardson E, Paxton JW, Baguley BC (2013). Tumour tissue selectivity in the uptake and retention of SN 28049, a new topoisomerase II-directed anticancer agent. Cancer Chemother Pharmacol.

[47] Parker AS, Eckel-Passow JE, Serie D, Hilton T, Parasramka M, Joseph RW, Wu KJ, Cheville JC, Leibovich BC (2014). Higher expression of topoisomerase II alpha is an independent marker of increased risk of cancer-specific death in patients with clear cell renal cell carcinoma. Eur Urol.

[48] Ramalingam D, Happel C, Ziegelbauer JM (2015). Kaposi's sarcoma-associated herpesvirus microRNAs repress breakpoint cluster region protein expression, enhance Rac1 activity, and increase *in vitro* angiogenesis. J Virol.

[49] Greco D, Kivi N, Qian K, Leivonen SK, Auvinen P, Auvinen E (2011). Human papillomavirus 16 E5 modulates the expression of host microRNAs. PloS one.

[50] Sahai E (2005). Mechanisms of cancer cell invasion. Curr Opin Genet Dev.

[51] Sanz-Moreno V, Gadea G, Ahn J, Paterson H, Marra P, Pinner S, Sahai E, Marshall CJ (2008). Rac activation and inactivation control plasticity of tumor cell movement. Cell.

[52] Vega FM, Ridley AJ (2008). Rho GTPases in cancer cell biology. FEBS Lett.

[53] Yamazaki D, Kurisu S, Takenawa T (2009). Involvement of Rac and Rho signaling in cancer cell motility in 3D substrates. Oncogene.

[54] Wang SM, Ooi LL, Hui KM (2011). Upregulation of Rac GTPase-activating protein 1 is significantly associated with the early recurrence of human hepatocellular carcinoma. Clin Cancer Res.

[55] Pliarchopoulou K, Kalogeras KT, Kronenwett R, Wirtz RM, Eleftheraki AG, Batistatou A, Bobos M, Soupos N, Polychronidou G, Gogas H, Samantas E, Christodoulou C, Makatsoris T (2013). Prognostic significance of RACGAP1 mRNA expression in high-risk early breast cancer: a study in primary tumors of breast cancer patients participating in a randomized Hellenic Cooperative Oncology Group trial. Cancer Chemother Pharmacol.

[56] Yeh CM, Sung WW, Lai HW, Hsieh MJ, Yen HH, Su TC, Chang WH, Chen CY, Ko JL, Chen CJ (2016). Opposing prognostic roles of nuclear and cytoplasmic RACGAP1 expression in colorectal cancer patients. Hum Pathol.

[57] Lu KH, Patterson AP, Wang L, Marquez RT, Atkinson EN, Baggerly KA, Ramoth LR, Rosen DG, Liu J, Hellstrom I, Smith D, Hartmann L, Fishman D (2004). Selection of potential markers for epithelial ovarian cancer with gene expression arrays and recursive descent partition analysis. Clin Cancer Res.

[58] Ma XJ, Salunga R, Tuggle JT, Gaudet J, Enright E, McQuary P, Payette T, Pistone M, Stecker K, Zhang BM, Zhou YX, Varnholt H, Smith B (2003). Gene expression profiles of human breast cancer progression. Proc Natl Acad Sci U S A.

[59] Rosty C, Sheffer M, Tsafrir D, Stransky N, Tsafrir I, Peter M, de Cremoux P, de La Rochefordiere A, Salmon R, Dorval T, Thiery JP, Couturier J, Radvanyi F (2005). Identification of a proliferation gene cluster associated with HPV E6/E7 expression level and viral DNA load in invasive cervical carcinoma. Oncogene.

