# Supplementary material for: Comparative evaluation of three proliferation markers, Ki-67, TOP2A, and RacGAP1, in bronchopulmonary neuroendocrine neoplasms: Issues and prospects
Source: Oncotarget. 2016 May 31;7(27):41959–73. doi: 10.18632/oncotarget.9747 (PMC5173108; doi:10.18632/oncotarget.9747)
Supplement: Supplementary file 1 [file oncotarget-07-41959-s001.pdf]

Comparative evaluation of three proliferation markers, Ki-67, TOP2A, and RacGAP1, in bronchopulmonary neuroendocrine neoplasms: Issues and prospects

Supplementary Materials

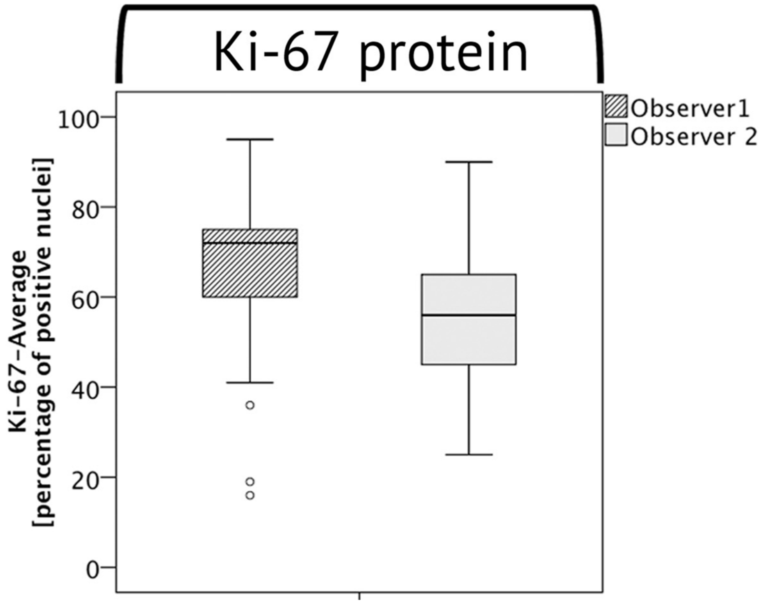

Supplementary Figure S1: Inter-observer variability of Ki-67 IHC. The boxplots are depicting the inter-observer variability: two observers independently investigated 22 randomly selected SCLC tumor samples by evaluating the Ki-67-Average by manual counting.

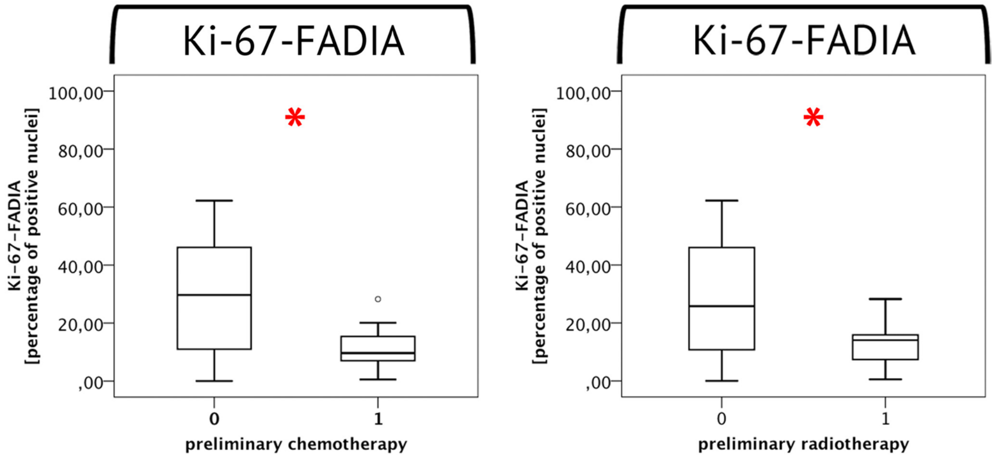

Supplementary Figure S2: Ki-67 expression vs. radiotherapy / chemotherapy. The boxplots are depicting the different Ki-67 expression levels of tumor samples before (0) and after (1) treatment with chemotherapy or radiotherapy as evaluated by means of Ki-67-FAIDA. Asterisks indicate significant differences according to the Mann-Whitney-Test.

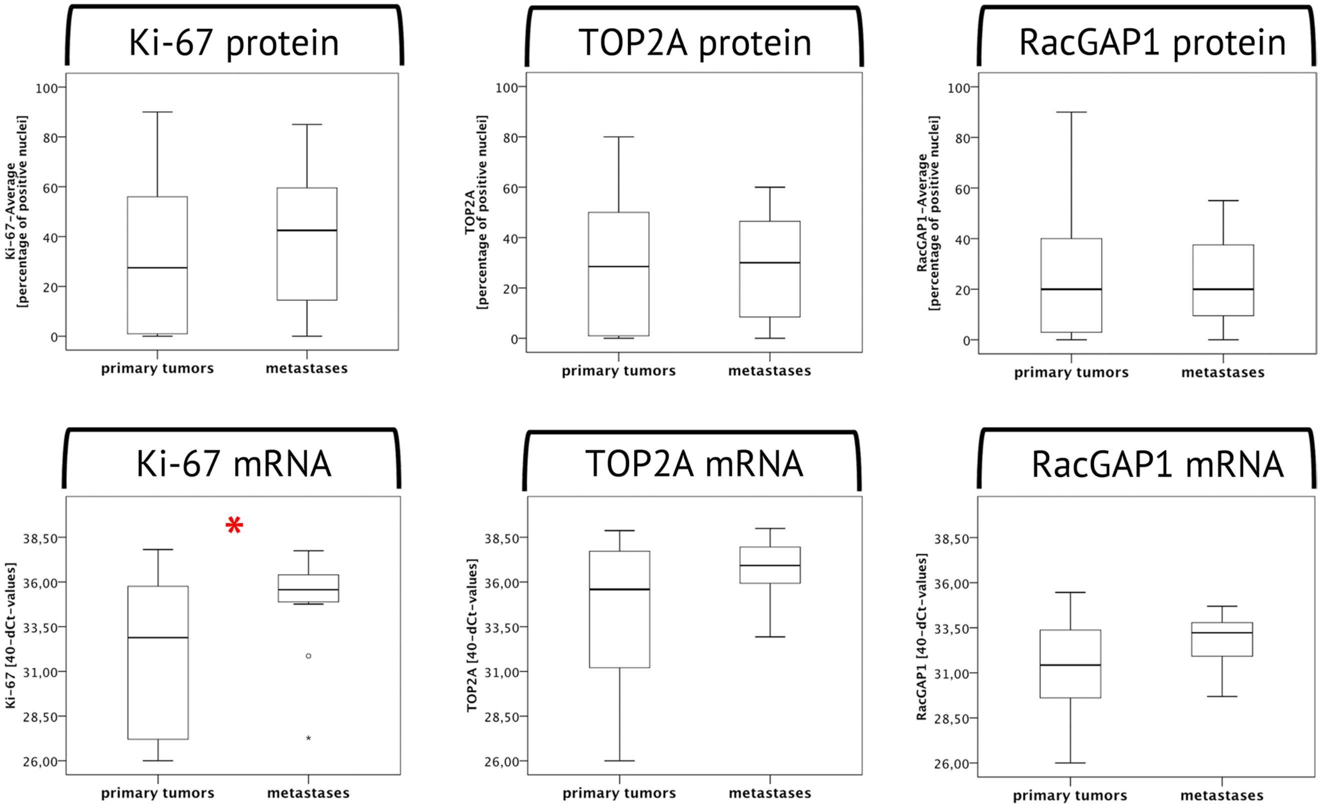

Supplementary Figure S3: Proliferation marker expression in primary tumors vs. metastases. The boxplots are depicting the protein (above) and mRNA (below) expression levels of Ki-67, TOP2A and RacGAP1 in primary tumors and metastases. Asterisks indicate significant differences according to the Mann-Whitney-Test.

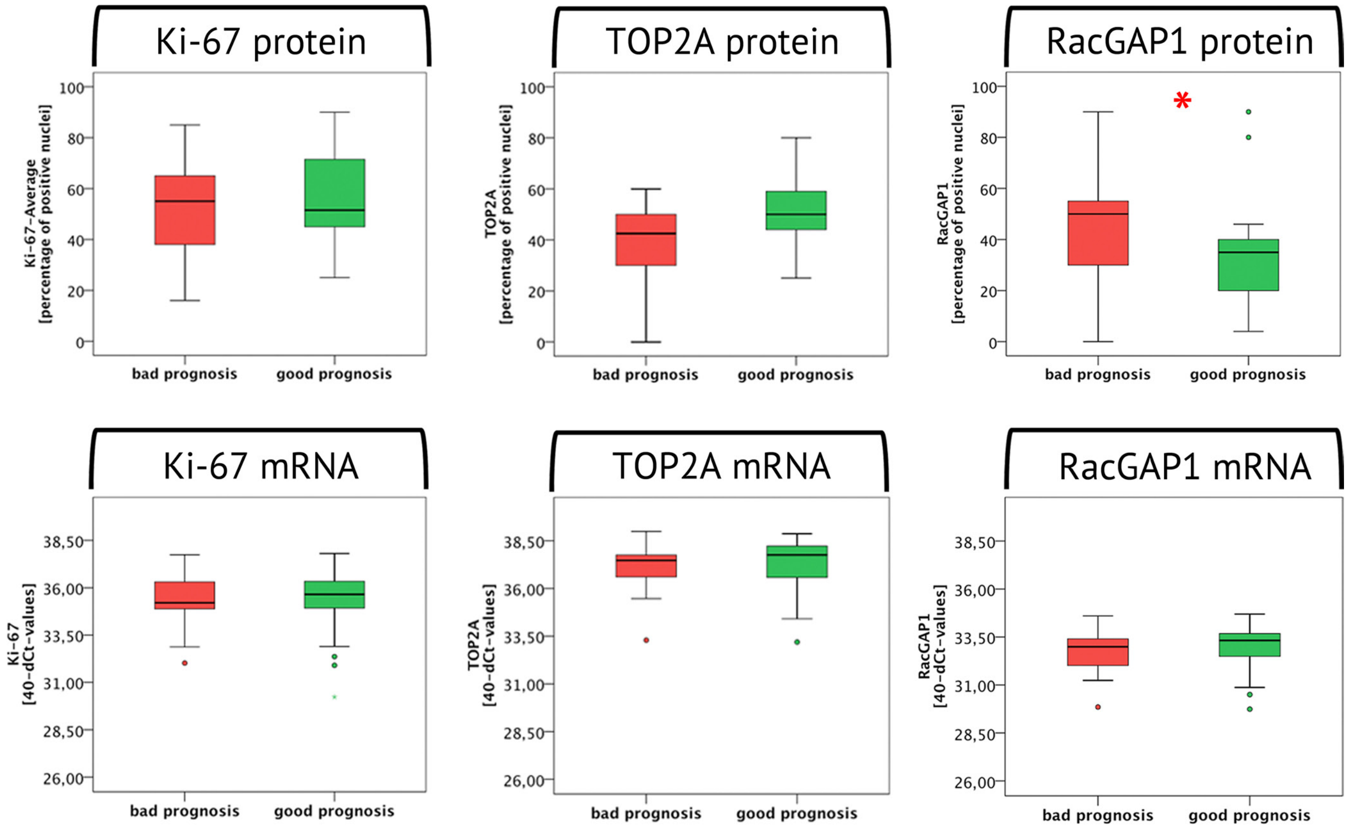

Supplementary Figure S4: Proliferation marker expression in SCLC with good and poor prognosis. The boxplots are depicting the protein and mRNA levels of Ki-67, TOP2A and RacGAP1 in SCLC with good prognosis (green, n = 28, survival ≥ 30 months) and poor prognosis (red, n = 21, survival < 30 months). Asterisks indicate significant differences according to the Mann-Whitney-Test.
